# Exploring the role of innate lymphoid cells in the periodontium: insights into immunological dynamics during orthodontic tooth movement

**DOI:** 10.3389/fimmu.2024.1428059

**Published:** 2024-07-03

**Authors:** Eva Pastille, Anna Konermann

**Affiliations:** ^1^ Institute of Medical Microbiology, University Hospital Essen, University of Duisburg-Essen, Essen, Germany; ^2^ Department of Orthodontics, University Hospital Bonn, Bonn, Germany

**Keywords:** innate lymphoid cells, mechanical strain, orthodontic tooth movement, periodontal ligament cells, sterile inflammation

## Abstract

**Background:**

The periodontal ligament (PDL) experiences considerable mechanical stresses between teeth and bone, vital for tissue adaptation, especially in orthodontic tooth movement (OTM). While recent research emphasizes the role of innate lymphoid cells (ILCs) in regulating sterile inflammation, their involvement in periodontal tissues during OTM remains largely unexplored.

**Methods:**

In this study, PDL tissues from orthodontic patients (*n* = 8) were examined using flow cytometry to detect ILC subtypes. Transwell co-culture systems were used to expose PDL cells to mechanical strain, followed by measuring migration and ratios of sorted ILC subtypes. Statistical analyses were conducted using paired Student’s t-test, Kruskal-Wallis test, Dunn’s post-test and one-way/two-way ANOVA with Tukey’s post-test (p≤ 0.05; **, p≤ 0.01; ***, p≤ 0.001).

**Results:**

Our findings demonstrate a significant increase in CD127+ CD161+ ILC frequencies in PDL tissues during OTM, indicating ILC involvement in sterile inflammation induced by orthodontic forces. Co-culture assays show directed migration of ILC subsets towards PDL cells and substantial proliferation and expansion of ILCs.

**Conclusions:**

This study is the first to comprehensively investigate the role of ILCs in sterile inflammation during OTM, revealing their presence and distribution within PDL tissues’ innate immune response *in vivo*, and exploring their migratory and proliferative behavior *in vitro*. The results suggest a crosstalk between ILCs and PDL cells, potentially influencing the inflammatory response and tissue remodeling processes associated with OTM.

## Introduction

The periodontal ligament (PDL) faces significant mechanical strains due to its location between teeth and bone, necessitating mechanisms to sustain cellular viability and equilibrium (1). Within this context, cellular networks endure physiological strains evoked by mastication, but also intense compressive and tensile forces generated during orthodontic tooth movement (OTM) (2). Specifically exerted orthodontic strains initiate a series of cellular and molecular responses, ultimately leading to tissue remodeling and adaptation, however always involving sterile inflammation (3). OTM is facilitated by the regulation of osteoclast-mediated bone resorption and osteoblast-mediated bone formation, with these remodeling processes significantly influenced by the immune system, orchestrating tooth displacement and associated tissue adaptations (4–6). Orthodontic force application triggering an inflammatory response leads to bone resorption on the compression side initiated by cytokines and chemokines, while osteogenic factors stimulate new bone formation on the tension side (7). The modulating influence of OTM and resulting mechanical strain impacts the immune microenvironment of periodontal tissues, causing alterations in the expression of cytokines and chemokines, involve signaling pathways facilitating the recruitment and activation of immune cells as much as the interaction between immune cells and host stromal cells to initiate inflammation and induce remodeling (3, 8, 9). Insufficient adaptation to mechanical stress may entail cell damage and tissue destruction, ultimately leading to root resorption as a harmful side effect of OTM, thus highlighting the need for a deeper understanding of the mechanisms protecting against these adverse effects in the mechanically active periodontal ligament (10). However, additional research is needed to uncover the precise mechanisms decisive on periodontal homeostasis versus inflammatory exacerbation.

Recent studies have shed light on the diverse functions of innate lymphoid cells (ILCs) and their subsets including ILC1s, ILC2s and ILC3s, emerging as crucial players in both acute and chronic immune regulation by orchestration of the delicate balance of tissue health and inflammation (11, 12). In contemporary characterization, ILCs have emerged as a distinct subset of immune cells devoid of recombination activating gene (RAG)-dependent rearranged antigen receptors as much as of myeloid cell and dendritic cell phenotypical markers (13). Typified by the expression of CD161, ILCs that are largely tissue-resident and deeply integrated into the fabric of tissues have been identified pivotal in orchestrating innate immune responses, maintaining tissue homeostasis and modulating inflammatory host defense (14). Classifiable into three principal subsets based on their distinctive cytokine secretion profiles, effector functions and transcription factor expression patterns, ILC1s have been identified as key effectors in combating intracellular pathogens, ILC2s are implicated in promoting tissue repair mechanisms and modulating allergic reactions, while ILC3s play critical roles in preserving mucosal barrier integrity and mounting responses against microbial challenges. ILC1 disclosing a Th1-like cytokine signature, are characterized by the T-box transcription factor Tbet and secrete interferon-gamma (IFN-g), while ILC2, exhibiting a Th2-like cytokine profile and typified by the transcription factors GATA3 and RORa primarily secrete type 2 cytokines such as IL-5 and IL-13, and finally ILC3, which are regulated by the transcription factor RORgt and have the ability to produce IL-17 and/or IL-22 (12). ILCs, with their rapid reactivity and ubiquitous distribution in healthy tissues, are crucial for facilitating communication between lymphoid and non-hematopoietic cells, playing essential roles in maintaining homeostasis and promoting tissue tolerance and regeneration in response to damage (15).

Furthermore, ILCs demonstrate diverse functions extending beyond their regulatory roles in tissue equilibrium, implicating participation in tissue remodeling, immune responses, and inflammatory processes, with recent investigations underscoring their involvement in bone remodeling and osteoimmunology (16). The engagement of ILCs in bone homeostasis, under both physiological and pathological conditions, implies interactions with osteoclasts and osteoblasts modulated by immunoregulatory networks orchestrated by ILC-derived cytokines and growth factors. At this, research indicates the integral contribution of all ILC subsets, including ILC1s producing RANKL to promote osteoclastogenesis, alongside ILC2s and ILC3s, in impacting both bone balance as much as pathological conditions, emphasizing their central role in osteoimmunology (17). Within bone and its surrounding tissues, ILCs possess the capability to produce and release various cytokines and growth factors, thereby exerting critical influence on osteoclast and osteoblast differentiation and activation processes, either facilitating bone resorption or formation (16).

The driving signals of sterile inflammation as found in OTM are recognized as damage-associated molecular patterns (DAMPs) (18). These entities, while distinct from their inflammatory pathogenic counterparts, exert their effects through many of the same signaling pathways and networks, whereas their pivotal role lies in orchestrating sterile inflammation by activating various cell types (19, 20). Recent research indicates that ILCs could be pivotal in inflammation without infection, reacting rapidly to innate stimuli without an apparent need for antigenic stimulation, which implies their potential significance as inflammation mediators in sterile environments. Intriguingly, evidence suggests these cells can assume both pro-inflammatory and pro-resolving functions, influenced by the inflammatory milieu and expressed cytokines (21). Despite understanding that ILCs play a role in sterile inflammatory responses across different tissues, their involvement in sterile inflammation within periodontal tissues remains uncertain. To our knowledge, only a limited number of studies have been published thus far highlighting the presence of ILCs in periodontal tissues, with two investigations indicating their role in immune response regulation in periodontitis (17, 22–24). However, research on the dynamics of ILCs during OTM and their specific impact on sterile inflammation induced by mechanical stress is still lacking.

This work aims to decipher whether and how ILCs are involved in local immune response of the PDL as a reaction to mechanical strain in the absence of microbial triggers caused by OTM, seeking to unravel the potential intricate interplay between ILCs and the periodontal microenvironment. Understanding the interplay between ILC subsets, mechanical strain and sterile inflammation in periodontal tissues could offer novel insights into therapeutic strategies for potential side effects of OTM, as understanding the complex dynamics of ILCs in periodontal tissues will undoubtedly contribute to the development of innovative treatment strategies aimed at restoring periodontal homeostasis.

## Materials and methods

The study was performed according to the ethical principles of the World Medical Association Declaration of Helsinki. Informed consent was obtained from all individual human donors of the experimental material included in the study. The study has been independently reviewed and approved by the Ethical Committee of the University of Bonn, Germany (reference number 029/08).

### Isolation of *in vivo* PDL specimens with and without exposure to OTM

*In vivo* PDL samples were derived from adolescent patients (*n* = 8, 2 male, 6 female, mean age 19.2 years ±6.8) starting orthodontic therapy with fixed multibracket appliances (022 slot size) and the therapeutic need for symmetric premolar extraction in one jaw. Patient selection criteria incorporated good general health, no medication affecting bone or soft tissue metabolism, no prosthetic restorations on the moved teeth, no premature occlusal contacts, no radiographic signs of horizontal bone loss or vertical bony defects and no manifestations of root resorptions. Given that brackets can serve as sites predisposed to plaque formation, it was imperative for all participants in the study to maintain optimal oral hygiene and receive precise oral hygiene instructions prior to bracket placement. This approach was crucial to eliminate any potential plaque-induced bacterial inflammation, thereby ensuring the integrity of the investigation results.

As part of the multibracket appliance insertion, one of the two premolars to be extracted was also fitted with a bracket for biomechanical reasons to optimize the movements of the neighboring teeth, whereas the contralateral premolar to be extracted was left out from the appliance remaining unbracketed. Thus, one of the two premolars was subjected to orthodontic loading during the leveling phase, while the other premolar served as a reference of physiological loading without OTM. After a period of 3-8 weeks depending on the therapeutic need, both premolars were extracted to gain therapeutic space and processed directly for further analyses.

After extraction and washing with PBS, the primary PDL tissue was explanted from the middle third of the tooth root surface after washing with PBS and the collected tissue was cut into small pieces in a petri dish. The tissue was digested in 500 µl RPMI medium containing 250 µg/mL Liberase (Roche, Basel, Switzerland) and 500 µg/mL DNAse (Sigma Aldrich, St. Louis, MO, USA) for 15 min. at 37°C under constant shaking. The digestion was stopped by placing the samples on ice and adding 1 mL RPMI medium supplemented with 2% FCS. The digestion mix was passed through a 70 µm cell strainer followed by centrifugation (5 min., 8°C, 1500 rpm). Then, specimens were directly stained for flow cytometric analyses.

### Flow cytometric analysis of ILCs in primary PDL tissues

Specimens (*n* = 8) were stained with antibodies (Biolegend, San Diego, CA, USA) against CD1a, CD14, CD19, CD20, CD34, CD123, CD303, NKp44, TCRαβ, TCRγδ and FcϵR1, as lineage markers, as well as anti-human CD45, CD127, CD161, CD117, CRTH2 and fixable viability dye eFluor™ 780 (Thermo Fisher Scientific). Labelled cells were sorted for ILC1s (viable CD45^+^ lin^-^ CD127^+^ CD161^+^ CD117^-^ CRTH2^-^), ILC2s (viable CD45^+^ lin^-^ CD127^+^ CD161^+^ CD117^-/+^ CRTH2^+^), and ILC3s (viable CD45^+^ lin^-^ CD127^+^ CD161^+^ CD117^+^ CRTH2^-^) (Vivier et al., 2018), using a FACSAria™ II cell sorter (BD Biosciences, San Jose, CA, USA).

### *In vitro* PDL cell culture

PDL tissues were explanted from the middle third of the root surface of teeth removed during routine extraction (*n* = 5, 1 male, 4 female, mean age 24.4 ± 23.4). Inclusion criteria are described above. Cells were grown in cell culture flasks (T75, CELLSTAR^®^ Greiner BioOne, Kremsmünster, Austria) in N2B27-PDLsf medium (25) at 37°C in a humidified 5% CO_2_ atmosphere and passaged after reaching confluence. Medium was supplemented with 1% Penicillin-Streptomycin (Gibco, Carlsbad, CA, USA) and 1% Plasmocin prophylactic (Invivogen, Toulouse, France) until passage 2. From passage 3, both media were used in the absence of Penicillin-Streptomycin and Plasmocin prophylactic. Culture expanded cells were utilized for analyses at passage 3 - 4. The passaging of cells was performed with StemPro Accutase (Gibco) for 5-10 minutes at 37°C and the dissociation was stopped by diluting the enzyme with medium. All conditions were assessed in duplicates.

### Mechanical loading of PDL cells

To explore the modulation and interaction of ILC subsets in the presence of mechanically loaded PDL cells (*n* = 2, with experiments conducted in duplicate using different donors for each setup, resulting in a total of *n* = 4), PDL cells were cultured to 80% confluence on Bioflex^®^ collagen type I-coated culture plates with silicone membrane flexible bottom wells and subsequently subjected to static tensile strain (BF-3001C; Flexcell International, Hillsborough, NC, USA). The plates were placed into a strain device (FX-6000T™ Tension System, Flexcell International) provided with a BioFlex baseplate with cylindrical post as loading platform in the dimensions of the flexible-bottom wells. The setup incorporates a computer-regulated bioreactor utilizing vacuum pressure and positive air pressure to administer a precisely defined and controlled static deformation to cells cultivated in monolayer. Following cell seeding into the plates and a 24-hour growth period before experimentation, continuous cell straining was executed at a level of 5% (26). The BioFlex baseplate, along with the loading stations and loading posts, was positioned within an incubator to maintain a humidified 5% CO2 atmosphere at 37° where cells were subjected to mechanical loading for 24 hours under these conditions. Subsequently, the plates were taken out from the setup and the strained cells were detached from the flexible membranes for further experimentation with StemPro Accutase (Gibco). Unstrained cells were used as controls in each experiment. Experimentations were performed in duplicate.

### Transwell co-culture migration and expansion assays of ILCs and mechanically strained versus unstrained PDL cells

PBMCs (*n* = 2, with experiments conducted in duplicate using different donors for each setup, resulting in a total of *n* = 4) were purified from buffy coats of healthy human donors (provided by the blood bank of the University of Bonn) in compliance with institutional review board protocols using Ficoll/Paque (Life Technologies, Inc., Carlsbad, CA, USA) density gradient centrifugation of heparinized blood. PBMCs were stained with antibodies (Biolegend, San Diego, CA, USA) against CD1a, CD14, CD19, CD20, CD34, CD123, CD303, NKp44, TCRαβ, TCRγδ and FcϵR1, as lineage markers, as well as anti-human CD45, CD127, CD161, CD117, CRTH2 and fixable viability dye eFluor™ 780. Labelled cells were sorted for ILC1s (viable CD45^+^ lin^-^ CD127^+^ CD161^+^ CD117^-^ CRTH2^-^), ILC2s (viable CD45^+^ lin^-^ CD127^+^ CD161^+^ CD117^-/+^ CRTH2^+^), and ILC3s (viable CD45^+^ lin^-^ CD127^+^ CD161^+^ CD117^+^ CRTH2^-^), using a FACSAria™ III cell sorter (BD Biosciences).

For transwell co-culture migration assays, PDL cells (*n* = 2, with experiments conducted in duplicate using different donors for each setup, resulting in a total of *n* = 4) were subjected to either 5% static mechanical strain or left untreated for 24 hours, followed by detachment and seeding in 24-transwell plates (Costar; Corning, NY, USA) after removal from Flexcell device. Concurrently, after 5h of PDL cell attachment, sorted ILC1, ILC2 or ILC3 cells derived from PBMCs of healthy donors were seeded in the upper chamber of the transwell system with a 5.0 µm pore size (6.5mm diameter, Polycarbonate Membrane; Costar) to allow migration into the lower chamber. After 20 hours of incubation, ILCs migrated into the lower chamber were harvested and cell numbers were quantified using a MACSQuant^®^ Analyzer (Miltenyi Biotec, Bergisch Gladbach, Germany). The percentage of migrated cells was calculated by dividing the number of ILCs at the bottom by the initial number of seeded ILCs. Negative controls included ILCs cultured without PDL cells at the bottom chamber.

For transwell co-culture expansion assays, strained and unstrained PDL cells were directly cultured in 24-transwell plates (Costar) after removal from Flexcell device for 24h. Subsequently, after 5h of PDL cell attachment, transwell inserts of 0.4µm pore size (6.5mm diameter, Polycarbonate Membrane; Costar) were placed into the wells and filled with medium containing all three ILC subsets, whereat the pore size allowed substance exchange between PDL cells and ILCs, but no migration of the ILCs. As negative control, ILCs were cultured alone in transwell chambers under the same conditions. After 7 days of incubation time, ILCs were harvested form the upper chamber and ILC1, 2 and 3 were determined using a BD FACS Canto Flow Cytometer (BD Biosciences).

### Statistical analysis

Statistical analyses were performed with the GraphPad Prism 10.2.2 software (GraphPad Software, San Diego, CA, USA). To test for normal distribution, D’ Agostino and Pearson omnibus normality test was used. Single comparison of normally distributed data was performed by paired Student’s t-test. As indicated in the figure legends, differences between means of more than two normally distributed groups were assessed by either one-way or two-way ANOVA followed by Tukey´s post-test and differences between means of more than two groups without normal distribution were assessed by Kruskal-Wallis test followed by Dunn´s post-test. All data were expressed as means ± standard error of the mean (SEM) and p-values less than 0.05 were considered to be statistically significant.

## Results

### ILCs are expanded within the PDL during OTM

**Figure 1:**
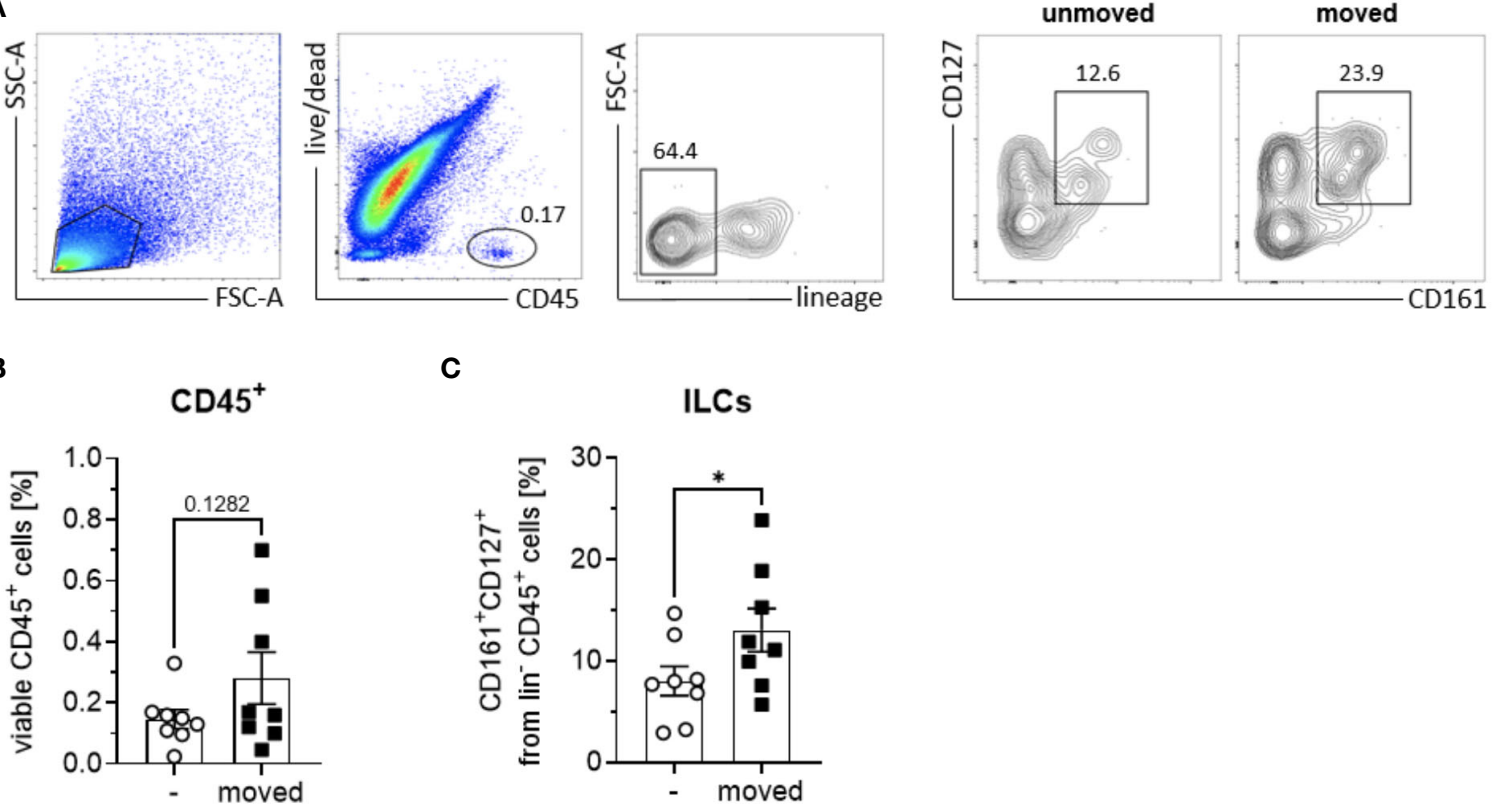
Quantification of ILCs in PDL tissues of orthodontically moved and unmoved teeth. From each patient (*n* = 8) undergoing orthodontic treatment, one control tooth and one moved tooth was extracted. PDL tissues were processed for detection of ILC subtypes via flow cytometric analysis, using a lineage marker antibody cocktail consisting of antibodies against human CD1a, CD14, CD19, CD20, CD34, CD123, CD303, NKp44, TCRαβ, TCRγδ and FcϵR1, as well as anti-human CD45, CD127, CD161, and fixable viability dye eFluor™ 780. **(A)** Total ILCs were gated as viable, CD45^+^ lin^-^ CD161^+^ CD127^+^ immune cells in PDL tissues from unmoved and moved teeth. Frequencies of **(B)** viable CD45^+^ immune cells and **(C)** CD127^+^ CD161^+^ ILCs among lin^-^ CD45^+^ immune cells. Graphs represent the mean ± SEM. Statistical significance was calculated using paired Student’s t test (*p≤ 0.05).

**Figure 2:**
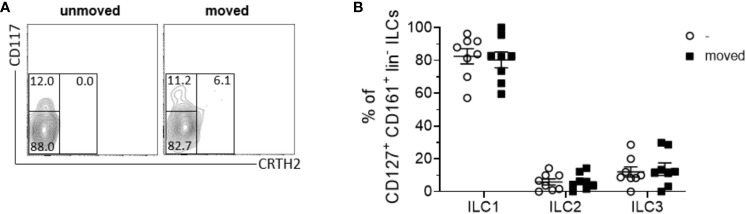
The composition of ILC subsets within the PDL remains unchanged following tooth movement. **(A)** Representative contour plot of CD117^-^ CRTH2^-^ ILC1s, CD117^-/+^ CRTH2^+^ ILC2s and CD117^+^ CRTH2^-^ ILC3s in PDL tissues from unmoved and moved teeth. **(B)** Scatter dot plots show the mean ± SEM of ILC1s, ILC2s and ILC3s in percent (*n =* 8).

Despite the recognized importance of ILCs in modulating innate immune responses across various tissues, their involvement in periodontal health and disease remains inadequately elucidated. Notably, research examining the presence and distribution of ILC subsets in sterile inflammation within the context of periodontal pathophysiology is currently lacking. In order to elucidate the role of ILCs in sterile inflammation occurring during OTM, PDL tissues obtained from both orthodontically moved and unmoved premolars subsequently extracted for therapeutic interventions some weeks after the start of multibracket appliance treatment, were subjected to analysis using flow cytometry to ascertain the frequencies of ILCs based on the expression levels of CD45, CD127, and CD161 markers (**Figure 1A**). A modest increase in CD45+ immune cell expansion was discernible in strained PDL tissues (0.3% ± 0.2) compared to unmoved controls (0.1% ± 0.1) (**Figure 1B**), however without displaying statistical significance (paired t-test, p=0.1282). Moreover, a notable augmentation in the frequencies of lineage marker negative CD127+ CD161+ ILCs among viable CD45+ cells was observed in the PDL derived from moved teeth (13.0% ± 6.0) when compared to unmoved teeth (8.0% ± 4.1), displaying statistical significance (paired t-test, p=0.0195) (**Figures 1A, C**). Analysis discerning the three distinct subsets of ILCs based on their expression of CRTH2 and CD117 demonstrated that OTM did not prompt the expansion of a particular ILC subset. Similar to unmoved teeth, ILCs from the PDL of moved teeth exhibited ILC1 (CD117^-^ CRTH2^-^) as the dominant subset, while ILC2 (CD117^-/+^ CRTH2^+^) and ILC3 (CD117^+^ CRTH2^-^) represented only smaller subsets (**Figures 2A, B**). Two-way ANOVA with Sidak post-test displayed p-values of p=0.9838 for ILC1, p=0.9990 for ILC2 and p=0.9717 for ILC3 and mean values for ILC1 of 82.5% ± 13.1 (unmoved) and 80.3% ± 13.7 (moved), for ILC2 of 5.9% ± 5.2 (unmoved) and 6.1% ± 4.9 (moved) and for ILC3 of 11.9% ± 8.7 (unmoved) and 13.7% ± 10.7 (moved).

### PDL cells induce migration of ILCs

**Figure 3:**
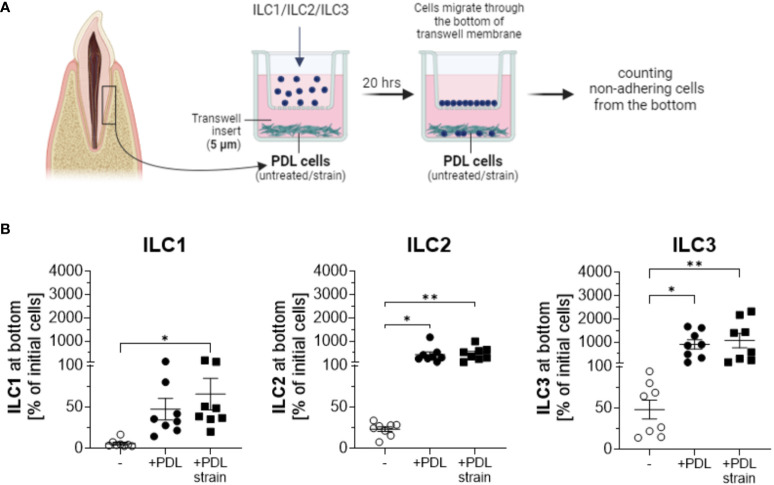
PDL cells induce migration and expansion of ILCs. PDL cells cultured with and without exposure to 5% static mechanical strain for 24 h were subsequently detached from wells and seeded in the bottom of transwell plates at 2 x 10^4^ cells per well for 5 h. Sorted ILC1, ILC2 or ILC3 cells from PBMCs were separately seeded in the upper chamber of the transwell system with 5.0 µm pore size to allow for migration into the lower chamber. Migration of each ILC population was measured after 20 h incubation. Therefore, non-adherent cells from the lower chamber were harvested and quantified by a MACSQuant^®^ Analyzer. **(A)** Graphical scheme of the experimental setup created with BioRender.com. **(B)** The percentage of migrated cells at the end of culture was calculated by dividing the number of ILCs at the bottom by the number of ILCs that were applied to the upper chamber at the beginning. ILCs cultured without PDL cells at the bottom chamber served as a negative control. The graphs show the mean ± SEM of data from 2 individual experiments with PDL cells and ILCs from 4 different donors. Statistical significance was calculated using Kruskal-Wallis test and Dunn´s post-test (for ILC1 and ILC2), or one-way ANOVA and Tukey`s post-test (for ILC3) (*p≤ 0.05; **p≤ 0.01).

Migration assays conducted using transwell culture systems with 5 µm pore sizes, allowing the migration of ILCs towards the lower chamber inhabiting PDL cells revealed that ILCs, irrespective of subtype ILC1, ILC2 or ILC3, migrated to the lower chamber (**Figure 3A**). The experiments employed ILC subsets in the same proportions per subgroup as they were quantitatively isolated from donors, reflecting their natural distribution.

In the absence of PDL cells in the lower chambers, some level of migration was observed, with ILC3 cells exhibiting the highest migration rates and ILC1 cells showing the lowest observed migration rates. Upon the addition of PDLs to the system, the migration of ILCs substantially intensified with statistical significance observed across all three ILC subtypes. Quantified cell numbers in the lower chamber exceeded the initial cell count in the upper chamber in several samples, suggesting not only migration of ILCs towards PDLs but also proliferation in their presence, a phenomenon not observed in the absence of PDL cells. Notably, ILC2s and ILC3s demonstrated substantial expansions, while the expansion of ILC1s was comparatively less pronounced. PDL cells subjected to mechanical strain exhibited a more pronounced increase in the attraction of ILC1s compared to untreated PDLs (**Figure 3B**). Analyses and statistics using Kruskal-Wallis test with Dunn´s post-test revealed that for ILC1, the numbers of migrated cells to the lower chamber were 5.9% ± 4.7 for controls without PDL cells, 47.4% ± 37.0 to unstrained PDL cells, and 65.6% ± 53.8 to strained PDL cells, with statistical significance observed for unstrained PDL cells compared to controls without PDL cell presence (p=0.0089) and strained PDL cells compared to controls without PDL cell presence (p=0.0006). For ILC2, analyses showed migration rates of 23.3% ± 8.3 for controls without PDL cells, 418.0% ± 326.9 to unstrained PDL cells, and 461.0% ± 280.8 to strained PDL cells, with statistical significance observed for both unstrained (p=0.0039) and strained (p=0.0011) PDL cell conditions compared to controls without PDL cells in the lower chamber (analyzed by Kruskal-Wallis test with Dunn´s post-test). Results for ILC3 featured values of 48.1% ± 32.1 of cells migrated to the lower chamber for controls without PDL cells, 905.7% ± 578.4 of cells to unstrained PDL cells and 1069.0% ± 881.1 of cells to strained PDL cells, again exhibiting statistical significance for both unstrained (p=0.0268) and strained (p=0.0081) PDL cell conditions compared to controls without PDL cells in the lower chamber (analyzed by one-way ANOVA with Tukey´s post-test) (**Figure 3B**).

When examining the individual migration percentages of ILCs in co-culture with PDL cells, a broad numerical range was observed for ILC1s, with migration rates spanning from approximately 20% to over 100%. In the case of ILC2s, the presence of PDL cells consistently induced significant proliferation, with maximum growth values exceeding 900%. Notably, except for two instances, this proliferation was further enhanced in the presence of mechanically loaded PDL cells. A similar trend was evident for ILC3s, wherein both extreme migration and population expansion were observed. The percentage increase in ILC3s was remarkably high, sometimes exceeding a thousand-fold, and on two occasions, even surpassing two thousand-fold in the presence of stretched PDL cells. Similar to the pattern observed with ILC2s, except for three instances, the increase in cell numbers was consistently more pronounced when expanded PDL cells were present compared to their absence. Absolute cell numbers of ILC subsets before and after co-culture as much as percentages are indicated in **Table 1**.

**Table 1:** PDL cell-induced migration/expansion of ILC subsets.

	Before culture (absolute cell numbers)*	After culture (absolute cell numbers)*			After culture (% of initial cells)*		
		no PDL	PDL control	PDL strain	no PDL	PDL control	PDL strain
**ILC1**	5500	278	802	2126	5.1	14.6	38.7
	5500	250	1954	2674	4.5	35.6	48.6
	5500	228	1794	1940	4.1	32.6	35.3
	5500	158	1526	2024	2.9	27.7	36.8
	702	118	294	1248	16.8	41.8	177.8
	702	54	562	822	7.7	80.1	117.1
	2509	70	3138	1270	2.8	125.1	50.6
	2509	90	548	504	3.6	21.8	20.1
**ILC2**	450	128	1456	2858	28.4	323.6	635.1
	450	126	2358	2572	28.0	524.0	571.6
	450	110	1114	1286	24.4	247.6	285.8
	450	96	1850	2362	21.3	411.1	524.9
	516	80	1320	1196	15.5	255.8	231.8
	516	40	592	550	7.8	114.7	106.6
	148	40	1732	1468	27.0	117.3	991.9
	148	50	440	504	33.8	297.3	340.5
**ILC3**	140	132	2262	3246	94.3	1615.7	2318.6
	140	112	2336	3038	80.0	1668.6	2170.0
	140	90	1202	1954	64.3	858.6	1395.7
	140	96	1780	2008	68.6	1271.4	1434.3
	172	38	1540	980	22.1	895.3	569.8
	172	46	558	504	26.7	324.4	293.0
	454	68	2186	1030	14.9	481.5	226.9
	454	64	590	642	14.1	129.9	141.4

*Cell numbers were obtained from two replicate experiments, each utilizing cells from two donors of ILCs, providing cells for two donors of PDL cells.

### PDL cells modulate the ILC subset proportions in culture

**Figure 4:**
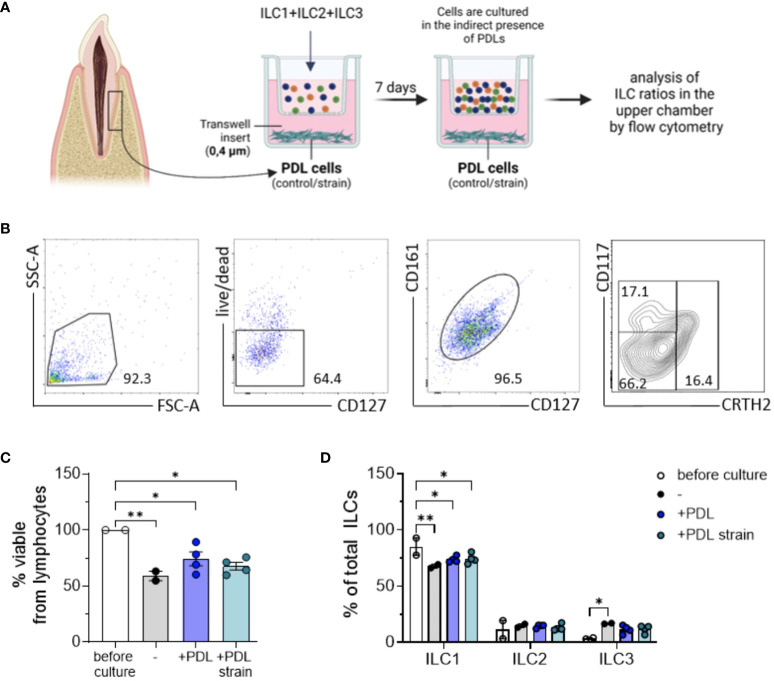
ILC expansion induced by PDL cells is contact-dependent. PDL cells cultured with and without exposure to 5% static mechanical strain for 24 h were subsequently detached from wells and seeded in the bottom of transwell plates at 2 x 10^4^ cells per well for 5 h. A mixture of sorted ILC1, ILC2 and ILC3 cells from PBMCs was pooled together and seeded in the upper chamber of the transwell system with 0.4 µm pore size to allow for substance exchange between PDL cells and ILCs, but no migration of the ILCs. After culture for 7 days, cells from the upper chamber were stained with antibodies against CD127, CD161, CD117, CRTH2 and fixable viability dye eFluor™ 780 and analyzed by flow cytometry. **(A)** Graphical scheme of the experimental setup created with BioRender.com. **(B)** Representative gating strategy to determine the frequencies of viable cells and CD117^-^ CRTH2^-^ ILC1s, CD117^-/+^ CRTH2^+^ ILC2s, and CD117^+^ CRTH2^-^ ILC3s. **(C)** Percentage of fixable viability dye eFluor™ 780 negative cells among lymphocytes. **(D)** Percentages of CD117^-^ CRTH2^-^ ILC1s, CD117^-^ CRTH2^+^ ILC2s, and CD117^+^ CRTH2^-^ ILC3s among CD127^+^ CD161^+^ ILCs. The graphs represent the mean ± SEM of one representative experiment with PDL cells and ILCs from 2 different donors each. Statistical significance was calculated using **(C)** one-wANOVA and Tukey`s post-test or **(D)** two-way ANOVA and Tukey`s post-test (*p≤ 0.05; **p≤ 0.01).

Analyses of ILC subset proportions in the presence of PDL cells, conducted using transwell culture systems equipped with 0.4 µm pore size inserts (**Figure 4A**), revealed that more than 50% of the acquired ILCs remained viable over a 7-day culture period (**Figures 4B, C**). The experimental assays employed ILC subsets in accordance with their natural proportions as isolated from the donors.

Compared to the initial composition of ILC cluster regarding its subset percentages, slight changes were observed after 7 days of culture. Analysis of PDL cell-induced modification of ILC subset ratios using two-way ANOVA along with Tukey posttest exhibited a statistically significant decrease of ILC1s after 7 days in culture, a reduction that was slightly attenuated when PDL cells were present in the lower compartments of the culture system. Prior to culture, the mean percentage of ILC1s among the three subsets was 85.1% ± 0.7, which decreased to 67.8 ± 2.2 relative to the other two subsets, namely ILC2 and ILC3, in the control group (p = 0.0026). In the presence of PDL cells, this decrease was observed to a lesser extent, with the percentage decreasing to 73.8 ± 2.7 (p = 0.0269) and furthermore to 74.4 ± 4.5 when PDL cells were subjected to mechanical strain (p = 0.0395). Contrarily, for ILC2 and ILC3 subsets, a moderate increase in cell ratios could be noted for all conditions in culture, however without reaching statistical significance (**Figure 4D**). ILC subset ratios before and after co-culture with and without mechanical strained PDL cells are presented in **Table 2**.

**Table 2:** PDL cell-induced modification of ILC subset ratios.

	Ratio before culture (%)		Ratio after culture (%)				
			no PDL	PDL control		PDL strain	
				PDL donor 1	PDL donor 2	PDL donor 1	PDL donor 2
ILC donor 1	**ILC1**	77.6	66.2	73.8	77.6	69.7	73.2
	**ILC2**	19.3	16.3	15.0	15.3	17.4	12.1
	**ILC3**	3.1	17.1	10.8	6.6	12.2	14.1
ILC donor 2	**ILC1**	92.7	69.3	72.1	71.5	74.2	80.5
	**ILC2**	3.3	13.3	12.1	15.2	11.0	11.8
	**ILC3**	4.0	16.4	15.3	13.1	14.3	6.8

## Discussion

The present study represents the first comprehensive investigation into the role of ILCs in sterile inflammation occurring during OTM, a process vital for orthodontic treatment but whose immunological underpinnings remain poorly understood. Specifically, our study aimed to elucidate the presence and distribution of ILC subsets within the innate immune response of PDL tissues undergoing OTM *in vivo*, and furthermore to explore their migratory and proliferative behavior in response to mechanically unstrained and strained PDL cells *in vitro*.

Our findings revealed a significant increase in the frequencies of CD127+ CD161+ ILCs in strained PDL tissues *in vivo* compared to unmoved controls, underscoring the involvement of ILCs in sterile inflammation induced by orthodontic forces. Thus, this study not only highlights the presence of ILCs in the periodontium but is the first work to demonstrate their dynamic response to mechanical strain, suggesting a potential role in modulating the immune microenvironment during OTM. At this, future investigations should investigate the underlying molecular mechanisms driving these sterile inflammatory processes. Specifically, IL-1β, renowned for its central involvement in various sterile inflammatory conditions in both innate and adaptive immunity, emerges as a prime candidate for targeted exploration (27). Previous studies have revealed elevated levels of IL-1β expression not only in endothelial cells and perivascular cells but also in fibroblasts throughout the PDL during orthodontic force application, reflecting a sterile inflammatory reaction (28). DAMPs, including IL-1ß, act as proinflammatory mediators predominantly activated in response to cellular stress or injury. Emerging evidence indicates that these DAMPs play a crucial role in modulating ILC subset responses, potentially exerting a significant influence on neutrophil recruitment in sterile inflammatory contexts (29). The present study’s findings align with these observations, highlighting the central role of PDL cells in potentially shaping the expansion of ILC subsets, which might be inter alia facilitated IL-1β secretion. Further investigations are warranted to thoroughly explore this relationship and explore the impact of immunomodulatory signaling by resident tissue-specific cells in sterile inflammation and its potential impact on the composition and function of ILCs.

As a secondary novel finding of this study, migration assays revealed that ILCs, irrespective of subtype, exhibited significantly increased migration towards PDL cells, especially when mechanically strained, though this increase did not reach statistical significance compared to unstrained PDL cells. This suggests a crosstalk between ILCs and PDL cells, potentially influencing the inflammatory response and tissue remodeling processes associated with OTM.

Notably, our study delineates distinct migratory and proliferative patterns among ILC subsets, with ILC1s, ILC2s, and ILC3s displaying differential responses to mechanical strain and PDL cell interactions. While ILC1s exhibited a significant increase in migration towards strained PDL cells, ILC2s and ILC3s demonstrated substantial expansion and proliferation, particularly in the presence of mechanically loaded PDL cells. These findings underscore the complexity of ILC biology and highlight the importance of considering subset-specific responses in understanding their role in sterile inflammation and tissue homeostasis during OTM.

Moreover, our results demonstrate the stability of ILC subsets over a 7-day culture period in the presence of PDL cells, with slight changes in subset proportions observed. Even though not statistically significant but obvious as a robust trend, the presence of PDL cells attenuated the decrease in ILC1 proportions observed in the absence of PDL cells, further emphasizing the regulatory role of PDL cells in modulating ILC subset ratios during OTM. Studies have indicated that while the phenotype of each ILC subset is clearly defined, environmental signals can induce the interconversion of phenotypes and the plasticity of ILCs through various external and internal factors (30). However, the alterations in cellular metabolism underlying ILC plasticity have been largely overlooked. Considering that metabolic shifts play a crucial role in determining the fate and function of these immune cells, the presence of PDL cells, along with sterile inflammation resulting from OTM, may emerge as newly recognized drivers for ILC phenotypic transitions, as observed in our study.

The outcomes of this study offer significant insights into the occurrence of ILCs and their immunomodulatory interactions within PDL tissues, however acknowledging limitations evolving from the methodology applied. This underscores the necessity of interpreting the conclusions in light of the chosen methodology while remaining cognizant of potential biases and the likelihood of obtaining different results with alternative experimental approaches. At this, one limitation encompasses the examination of one single time point for readouts and the utilization of one strain intensity. Other studies have demonstrated that in sterile inflammation, substantial differences in experimental outcomes can arise between low- and high-intensity sterile inflammation scenarios (31).

Additionally, the generalizability of the results may be constrained by the unique characteristics of the donors studied, encompassing potential variations among donors that could influence cellular processes. This necessitates careful extrapolation beyond the current dataset to ensure unbiased and comprehensive conclusions. Specifically, the different treatment durations and extraction time points varying between 3-8 weeks and necessitated by the diverse treatment requirements of the participants, could lead to differences in ILC expression phenotypes. However, our *in vivo* data analysis demonstrated strong concordance among the different donors in this regard, suggesting minimal influence of temporal differences within the context of our individualized treatment durations. Nonetheless, these limitations should be considered when interpreting the data on a broader scale. Furthermore, it is imperative to emphasize the necessity for further validation across a wider range of *in vivo* and *in vitro* samples to establish the functional relevance of our findings. Finally, subsequent research ought to prioritize the thorough examination of gene and protein expression patterns of immunomodulatory molecules within both ILCs and PDL cells. Future investigations should analyze the precise interplay between these molecules and their impact on the process of OTM as well as the underlying immunological cascades orchestrating this phenomenon in order to enhance the understanding of the molecular mechanisms governing OTM and to potentially unveil novel therapeutic targets for optimizing orthodontic treatment outcomes.

Our study provides novel and unprecedented insights into the involvement of ILCs in sterile inflammation during OTM, highlighting their dynamic behavior in response to mechanical strain and PDL cell interactions. By elucidating the role of ILC subsets in modulating the immune microenvironment of the periodontium, our findings pave the way for further research aimed at targeting ILCs as potential therapeutic targets for managing inflammation and promoting tissue homeostasis during orthodontic treatment.

In conclusion, this study sheds light on the previously unexplored role of ILCs and the corresponding subsets ILC1s, ILC2s and ILC3s in sterile inflammation during OTM both *in vivo* and *in vitro*. By investigating the presence and behavior of ILC subsets in mechanically strained PDL tissues, the findings reveal significant increases in CD127+ CD161+ ILCs, emphasizing their involvement in sterile inflammation induced by orthodontic forces. Furthermore, the study uncovers a dynamic interplay between ILCs and PDL cells, with distinct migratory and proliferative patterns observed among ILC subsets. These insights offer promising avenues for future research targeting ILCs as potential therapeutic targets for managing inflammation and promoting tissue homeostasis during orthodontic treatment.

## Data availability statement

The original contributions presented in the study are included in the article/supplementary materials, further inquiries can be directed to the corresponding author/s.

## Ethics statement

The studies involving humans were approved by Ethical committee of the University of Bonn. The studies were conducted in accordance with the local legislation and institutional requirements. Written informed consent for participation in this study was provided by the participants or the participants’ legal guardians/next of kin.

## Author contributions

EP: Data curation, Formal analysis, Methodology, Writing – review & editing. AK: Conceptualization, Project administration, Supervision, Validation, Writing – original draft.
